# *Chlamydophila abortus* Pelvic Inflammatory Disease

**DOI:** 10.3201/eid0912.020566

**Published:** 2003-12

**Authors:** Gernot Walder, Herwig Meusburger, Helmut Hotzel, Albrecht Oehme, Walter Neunteufel, Manfred P. Dierich, Reinhard Würzner

**Affiliations:** * Institute of Hygiene and Social Medicine, University of Innsbruck, Innsbruck, Austria; † District Hospital of Dornbirn, Dornbirn, Austria; ‡ Federal Institute for Consumer Protection and Veterinary Medicine, Jena, Germany; § Friedrich-Schiller-Universität Jena, Jena, Germany

**Keywords:** *Chlamydophila abortus*, pelvic inflammatory disease, ompA-gene, stress-response protein, hsp60, intrauterine device, copper

## Abstract

We report the first documented case of an extragestational infection with *Chlamydophila abortus* in humans. The pathogen was identified in a patient with severe pelvic inflammatory disease (PID) by sequence analysis of the *ompA* gene. Our findings raise the possibility that *Chlamydiaceae* other than *Chlamydia trachomatis* are involved in PID.

*Chlamydophila (Cp.) abortus*, whose strains are nearly 100% conserved in ribosomal and *ompA* genes, has recently been derived as new species from *Cp. psittaci* (*1*). It is the causative agent of enzoonotic abortion, which is frequently observed among sheep flocks in the eastern Alps and worldwide (*2*). By producing spontaneous abortion, stillbirth, or delivery of weak lambs, it is a major cause of reproductive failure in most sheep-rearing countries and, consequently, a serious economic problem (*3*).

*Cp. abortus* has also been characterized by serologic testing or sequence analysis from abortion in a horse, rabbit, pig, guinea pigs, and mice (*1*). It was first isolated from products of a septic human abortion in 1967 (*4*). Previously, human infections have been reported anecdotally (*5*), and *Cp. abortus* has been confirmed as the causative agent of septic abortion by ultrastructural and genetic analysis of isolates from women with previous contact with sheep (*6*–*8*). In humans, extragestational manifestations of infection with *Cp. abortus* have never been described. We therefore report the case of a 39-year-old woman with severe pelvic inflammatory disease (PID) caused by *Cp. abortus*.

## Case Report

In February 2001, a 39-year-old woman was admitted to the district hospital of Dornbirn, Vorarlberg, Austria, for chronic abdominal pain, increased vaginal discharge, and unusually heavy menses. Her medical record showed two uncomplicated pregnancies, followed by an aseptic abortion in the second trimester, a further uncomplicated pregnancy, and two first-trimester miscarriages. Since adolescence, the patient had experienced lower abdominal complaints, including menstrual irregularities, urinary symptoms, and unspecific vaginal discharge. Repeated treatment of mycosis and infections of the urinary tract did not lead to substantial clinical improvement nor did symptomatic treatment with corticosteroids or with *Lactobacillus acidophilus*. Her condition was exacerbated after a copper-containing intrauterine device was inserted in 1999. Episodes of lower abdominal pain became more frequent and more severe. They were accompanied by fatigue, general malaise, and sometimes by elevated temperature. Her menses became increasingly heavy, finally resulting in 10 days of heavy bleeding. Clinical investigation on admission showed lower abdominal tenderness, cervical motion tenderness, and bilateral adnexal tenderness, more prominent on the right. The patient had signs of compensatory hypochromic anemia, which was attributable to menorrhagia. Erythrocyte sedimentation rate (22–44 mm/h) and C-reactive protein (0.6 mg/dL) were moderately elevated. Leukocyte count was normal as was the patient’s oral temperature. Results of tests for *Neisseria gonorrhoeae* and *Chlamydia trachomatis* by ligase chain reaction (LCx; Abbot Laboratories, Vienna, Austria) were negative.

Because chronic PID was suspected, the intrauterine device was removed. Diagnostic pelviscopy indicated diffuse coalescence of both adnexes and edematous swelling of the fallopian tubes with severe postinflammatory changes (Figure). Amber liquid was extracted from the pouch of Douglas for further microbiologic investigation. Aerobic and anaerobic cultures remained sterile as did cultures for *Ureaplasma urealyticum* and *Mycoplasma hominis* on special media (Biomerieux, Nürtingen, Germany). Results of LCx tests for *C. trachomatis* and *N. gonorrhoeae* were negative, and notable levels of antibodies against a constant region of the major outer membrane protein of *C. trachomatis* were not found in the patient’s serum by enzyme-linked immunosorbent assay (ELISA) (Medac, Hamburg, Germany). However, the patient showed high levels of antibodies against genus-specific lipopolysaccharide of *Chlamydiacaea* (LPS-ELISA, Medac). Thus, microimmunofluorescence assays (MIF) for *C. trachomatis*, *Cp. pneumoniae* (both in house MIF Jena) and *Cp. psittaci* (Biomerieux, Nürtingen, Germany) showed high antibody titers against *C. psittaci* (1:512), titers against *Cp. pneumoniae* were within the normal range (1:16), and the result of the *C. trachomatis* reaction was unspecific and interpreted as negative. A retrospective analysis found that notable levels of antibodies against the heat-shock protein 60 (hsp60) were demonstrated in the patient’s serum by ELISA. Nested polymerase chain reaction (PCR) for *Chlamydiaceae* spp.*–*specific *ompA* (*9*) was done from the pouch of Douglas liquid and yielded a positive result.

**Figure:**
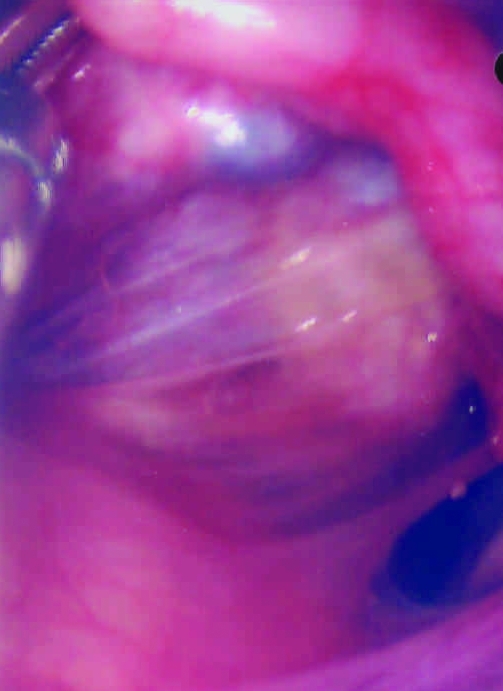
Diffuse coalescences between uterus and fallopian tubes (the ovary is hidden behind the uterus).

Sequence analysis of the resulting PCR product showed that it had the highest homology to *Cp. abortus*. The isolate’s *ompA* gene region was >99% homologous with *Cp. abortus* strains, whereas *Cp. psittaci* was <90% homologous.

The patient was treated with doxycycline (Vibravenoes, 200 mg–100 mg for 5 days). Six weeks later, all laboratory parameters were within normal ranges, the patient’s fatigue had subsided, and she had not experienced further episodes of elevated temperature. Tenderness of the lower abdomen had subsided, except for a slight monolateral adnexal tenderness on the right side, consistent with a decreased swelling of the right fallopian tube shown by sonogram. Eighteen weeks later, both adnexes and the lower abdomen were indolent upon palpation, the patient felt well, and a sonogram showed a further regression of the swelling of the right fallopian tube. When questioned 9 months after treatment, the patient did not report any lower abdominal symptoms, and her menstrual irregularities had subsided.

## Discussion

The patient had likely been infected with *Cp. abortus*. This conclusion is supported by the positive result of the PCR from Douglas liquid and the sequence analysis of the *ompA* gene. Because *Cp. abortus* is highly infectious, it requires C3 equipment for culturing; thus, no attempt was made to confirm this result by culture. The serologic investigation provided further support for the involvement of *Cp. abortus* in this case: High antibody titers to LPS indicate chronic infection or, less likely, multiple expositions to a member of the genus *Chlamydiaceae* (*10*). The high immunofluorescence assay titer to *Cp. psittaci* is consistent with an infection with *Cp. abortus* because both species are closely related and share most surface proteins (*11*), making a serologic distinction between both pathogens virtually impossible. The absence of antibodies specific to *C. trachomatis* and the repeatedly negative results of the ligase chain reaction for detection of *C. trachomatis* exclude a concomitant infection with this pathogen. A careful search for other microorganisms in the patient’s Douglas extract did not yield a pathologic result.

Infection with *Cp. abortus* has hitherto exclusively been reported in pregnant women, beginning as an influenzalike illness with consecutive development of thrombocytopenia and coagulopathy, usually resulting in fetal death (*4*,*5*). Symptomatic carriers have been described in sheep, with the pathogen being shed in periovulatory estrus (*12*), but the possibility of chronic infection or the possibility of extragestational illness has never been evaluated in humans.

In light of previous investigations of chronic infection with *C. trachomatis,* *Cp. abortus* appears to be a probable PID. Salpingitis and postinflammatory adhesions as observed in our patient are known sequelae of genital chlamydial infection in animals and humans (*13*). Some evidence shows that inflammation and subsequent tissue damage in chronic PID are due to an immunopathologic reaction against a chlamydial heat-shock protein (hsp60) (*14*). Heat-shock proteins are highly conservative. A high amino acid identity exists between the hsp60 of *C. trachomatis* and the hsp60 of other *Chlamydiaceae* (*Cp.a* *caviae,* 93%; *Cp. pneumoniae*, 80%) as well as stress response proteins found in other microorganisms (*15*). Identity to the htpB protein of *Coxiella burnetii* is 61%, to the GroEL protein of *Escherichia coli* it is 60%, and to human HuCha 60, 48% (*15*). That the hsp60-specific antibodies in our patient’s serum were induced by *Cp. abortus* is highly likely, and this genus can cause PID in a way similar to that proposed for *C. trachomatis*. Thus, we conclude that *Cp. abortus* has to be considered in patients with PID disease and should be ruled out with suitable diagnostic methods. When PCR is applied, the preferred method should amplify sequences shared by all members of *Chlamydiaceae*.

The exacerbation of our patient’s symptoms after the insertion of the intrauterine device was striking, and the possible underlying mechanism needed to be considered. A coincidental infection with the pathogen at the same time was unlikely because no evidence for that was found in the patient’s medical history, and the serologic results pointed towards chronic infection. However, the patient reported extensive contact with sheep and other ruminants in her youth, which she terminated due to bronchopulmonary complaints. Whether they were due to an allergic reaction or an infection of the lower airways remained unclear. Her unspecific genecologic symptoms started at approxymately the same time, about 2 years after her menarche and about 5 years before her first pregnancy. Exact data on how the expression of hsp60 in *Chlamydia* is influenced by copper are not available. Limited evidence has indicated that copper induces the expression of hsp60 in rotifers (*16*) and that copper deficiency reduces the expression of hsp60-like proteins in rats (*17*). Because the amino acid sequence of the heat-shock protein and the amino acid sequence of the promotor region are highly conservative (*15*), we propose that the intrauterine device might have induced the expression of hsp60, which became the source of antigenic stimulation for an autopathologic immune response. Consistent with this theory is the finding that our patient’s symptoms subsided after the intrauterine device was removed and the chlamydial infection had been treated with antimicrobial drugs. Some evidence indicated that the chlamydial organisms are required in order for chronic PID to develop (*18*), and although information about the use of antimicrobial drugs in chronic stages of PID is limited, they have proven effective in other *Chlamydia*-triggered autoimmune diseases (*19*). Further studies are under way to investigate the clinical importance of extragestational infection with *C. abortus* and the influence of copper on the expression of stress response proteins in *Chlamydiaceae*.
